# Supplementary material for: Multi‐dataset identification of innovative feature genes and molecular mechanisms in keratoconus
Source: J Cell Mol Med. 2024 Sep 19;28(18):e70079. doi: 10.1111/jcmm.70079 (PMC11412914; doi:10.1111/jcmm.70079)
Supplement: Supplementary file 2 — Material S2. [file JCMM-28-e70079-s002.zip › 7.GSEA analysis/6.SNORA74B/KEGG_SPLICEOSOME.html]

Details for gene set KEGG\_SPLICEOSOME[GSEA]

|  || Dataset | SNORA74B.group.cls#h\_versus\_l |
| Phenotype | group.cls#h\_versus\_l |
| Upregulated in class | h |
| GeneSet | KEGG\_SPLICEOSOME |
| Enrichment Score (ES) | 0.15202242 |
| Normalized Enrichment Score (NES) | 0.64453304 |
| Nominal p-value | 0.93083006 |
| FDR q-value | 1.0 |
| FWER p-Value | 1.0 |
Table: GSEA Results Summary

  

Fig 1: Enrichment plot: KEGG\_SPLICEOSOME      
 Profile of the Running ES Score & Positions of GeneSet Members on the Rank Ordered List

  

| SYMBOL | TITLE | RANK IN GENE LIST | RANK METRIC SCORE | RUNNING ES | CORE ENRICHMENT || 1 | THOC1 | na | 2861 | 0.309 | -0.0390 | Yes |
| 2 | ACIN1 | na | 5315 | 0.215 | -0.0796 | Yes |
| 3 | PRPF40A | na | 5676 | 0.203 | -0.0613 | Yes |
| 4 | PHF5A | na | 8356 | 0.128 | -0.1207 | Yes |
| 5 | HSPA2 | na | 8508 | 0.124 | -0.1074 | Yes |
| 6 | SRSF6 | na | 8593 | 0.122 | -0.0925 | Yes |
| 7 | HSPA8 | na | 8644 | 0.121 | -0.0768 | Yes |
| 8 | RBM25 | na | 9091 | 0.111 | -0.0740 | Yes |
| 9 | SART1 | na | 9236 | 0.108 | -0.0628 | Yes |
| 10 | TRA2A | na | 9467 | 0.103 | -0.0548 | Yes |
| 11 | CDC5L | na | 9783 | 0.097 | -0.0502 | Yes |
| 12 | THOC2 | na | 10140 | 0.090 | -0.0478 | Yes |
| 13 | ISY1 | na | 10270 | 0.088 | -0.0392 | Yes |
| 14 | U2AF1 | na | 10996 | 0.075 | -0.0495 | Yes |
| 15 | SRSF1 | na | 11033 | 0.074 | -0.0400 | Yes |
| 16 | PRPF19 | na | 11204 | 0.071 | -0.0349 | Yes |
| 17 | SRSF3 | na | 11234 | 0.071 | -0.0257 | Yes |
| 18 | SF3B3 | na | 11571 | 0.066 | -0.0261 | Yes |
| 19 | XAB2 | na | 11963 | 0.061 | -0.0288 | Yes |
| 20 | HNRNPA1L2 | na | 12567 | 0.052 | -0.0388 | Yes |
| 21 | CDC40 | na | 12598 | 0.052 | -0.0324 | Yes |
| 22 | SF3B1 | na | 12921 | 0.048 | -0.0349 | Yes |
| 23 | NCBP1 | na | 13287 | 0.044 | -0.0393 | Yes |
| 24 | SRSF8 | na | 13690 | 0.039 | -0.0453 | Yes |
| 25 | CRNKL1 | na | 13772 | 0.039 | -0.0422 | Yes |
| 26 | PLRG1 | na | 14487 | 0.030 | -0.0586 | Yes |
| 27 | SRSF5 | na | 14607 | 0.029 | -0.0579 | Yes |
| 28 | HNRNPU | na | 14874 | 0.026 | -0.0620 | Yes |
| 29 | CWC15 | na | 14928 | 0.025 | -0.0600 | Yes |
| 30 | SRSF2 | na | 14942 | 0.025 | -0.0568 | Yes |
| 31 | HNRNPA1 | na | 15393 | 0.021 | -0.0669 | Yes |
| 32 | SNRNP70 | na | 15754 | 0.017 | -0.0749 | Yes |
| 33 | MAGOHB | na | 16211 | 0.013 | -0.0863 | Yes |
| 34 | CCDC12 | na | 16360 | 0.011 | -0.0890 | Yes |
| 35 | HSPA1A | na | 16403 | 0.010 | -0.0888 | Yes |
| 36 | PRPF6 | na | 16480 | 0.010 | -0.0896 | Yes |
| 37 | EFTUD2 | na | 16599 | 0.008 | -0.0918 | Yes |
| 38 | LSM7 | na | 16629 | 0.008 | -0.0915 | Yes |
| 39 | HNRNPA3 | na | 16934 | 0.005 | -0.0996 | Yes |
| 40 | HSPA1B | na | 17184 | 0.003 | -0.1063 | Yes |
| 41 | ZMAT2 | na | 17253 | 0.002 | -0.1080 | Yes |
| 42 | LSM3 | na | 17430 | 0.000 | -0.1130 | Yes |
| 43 | DHX8 | na | 17508 | -0.001 | -0.1152 | Yes |
| 44 | PRPF40B | na | 17542 | -0.001 | -0.1160 | Yes |
| 45 | SRSF9 | na | 17740 | -0.003 | -0.1212 | Yes |
| 46 | RBM22 | na | 17793 | -0.004 | -0.1222 | Yes |
| 47 | U2AF2 | na | 17856 | -0.004 | -0.1234 | Yes |
| 48 | ALYREF | na | 17987 | -0.006 | -0.1263 | Yes |
| 49 | SF3B5 | na | 18111 | -0.007 | -0.1289 | Yes |
| 50 | MAGOH | na | 18237 | -0.009 | -0.1313 | Yes |
| 51 | SF3A1 | na | 18377 | -0.011 | -0.1338 | Yes |
| 52 | SNRPF | na | 18553 | -0.012 | -0.1371 | Yes |
| 53 | SNRPG | na | 18560 | -0.012 | -0.1355 | Yes |
| 54 | TRA2B | na | 18621 | -0.013 | -0.1354 | Yes |
| 55 | RBM17 | na | 18767 | -0.015 | -0.1376 | Yes |
| 56 | SF3A2 | na | 18952 | -0.016 | -0.1406 | Yes |
| 57 | SNRPD1 | na | 18985 | -0.017 | -0.1391 | Yes |
| 58 | LSM8 | na | 19039 | -0.017 | -0.1382 | Yes |
| 59 | HNRNPK | na | 19132 | -0.019 | -0.1382 | Yes |
| 60 | PRPF8 | na | 19275 | -0.021 | -0.1394 | Yes |
| 61 | RBM8A | na | 19335 | -0.021 | -0.1381 | Yes |
| 62 | THOC3 | na | 19497 | -0.023 | -0.1395 | Yes |
| 63 | BUD31 | na | 19558 | -0.024 | -0.1379 | Yes |
| 64 | SNRPD2 | na | 19566 | -0.024 | -0.1348 | Yes |
| 65 | DDX39B | na | 19671 | -0.025 | -0.1343 | Yes |
| 66 | U2SURP | na | 19767 | -0.026 | -0.1333 | Yes |
| 67 | PRPF18 | na | 19802 | -0.026 | -0.1306 | Yes |
| 68 | TXNL4A | na | 19898 | -0.027 | -0.1295 | Yes |
| 69 | SRSF10 | na | 19903 | -0.027 | -0.1257 | Yes |
| 70 | LSM5 | na | 19937 | -0.028 | -0.1228 | Yes |
| 71 | PRPF4 | na | 20124 | -0.030 | -0.1239 | Yes |
| 72 | CTNNBL1 | na | 20325 | -0.032 | -0.1252 | Yes |
| 73 | DDX5 | na | 20346 | -0.032 | -0.1213 | Yes |
| 74 | SNRPE | na | 20371 | -0.032 | -0.1174 | Yes |
| 75 | SNRPB2 | na | 20408 | -0.033 | -0.1138 | Yes |
| 76 | SF3B6 | na | 20483 | -0.034 | -0.1111 | Yes |
| 77 | DDX42 | na | 20500 | -0.034 | -0.1068 | Yes |
| 78 | DDX46 | na | 20532 | -0.034 | -0.1028 | Yes |
| 79 | NCBP2 | na | 20541 | -0.035 | -0.0982 | Yes |
| 80 | PPIH | na | 20613 | -0.035 | -0.0952 | Yes |
| 81 | SF3B2 | na | 20686 | -0.036 | -0.0922 | Yes |
| 82 | SRSF4 | na | 20760 | -0.037 | -0.0890 | Yes |
| 83 | CHERP | na | 20980 | -0.040 | -0.0898 | Yes |
| 84 | DDX23 | na | 21118 | -0.041 | -0.0879 | Yes |
| 85 | SNRNP200 | na | 21422 | -0.045 | -0.0902 | Yes |
| 86 | HNRNPC | na | 21453 | -0.045 | -0.0847 | Yes |
| 87 | HNRNPM | na | 21596 | -0.047 | -0.0822 | Yes |
| 88 | LSM4 | na | 21745 | -0.049 | -0.0796 | Yes |
| 89 | USP39 | na | 21857 | -0.050 | -0.0757 | Yes |
| 90 | SNRPA | na | 21907 | -0.051 | -0.0700 | Yes |
| 91 | PRPF31 | na | 21937 | -0.051 | -0.0636 | Yes |
| 92 | LSM2 | na | 22026 | -0.052 | -0.0588 | Yes |
| 93 | PPIE | na | 22075 | -0.053 | -0.0527 | Yes |
| 94 | SNRNP40 | na | 22277 | -0.055 | -0.0507 | Yes |
| 95 | PCBP1 | na | 22383 | -0.057 | -0.0458 | Yes |
| 96 | PRPF38B | na | 22451 | -0.058 | -0.0396 | Yes |
| 97 | PUF60 | na | 22468 | -0.058 | -0.0319 | Yes |
| 98 | LSM6 | na | 22541 | -0.059 | -0.0257 | Yes |
| 99 | DHX16 | na | 22588 | -0.060 | -0.0186 | Yes |
| 100 | DHX38 | na | 22663 | -0.060 | -0.0122 | Yes |
| 101 | SNW1 | na | 22690 | -0.061 | -0.0044 | Yes |
| 102 | WBP11 | na | 22709 | -0.061 | 0.0037 | Yes |
| 103 | SLU7 | na | 22761 | -0.062 | 0.0110 | Yes |
| 104 | SF3A3 | na | 22850 | -0.063 | 0.0173 | Yes |
| 105 | AQR | na | 22859 | -0.063 | 0.0260 | Yes |
| 106 | EIF4A3 | na | 23017 | -0.066 | 0.0307 | Yes |
| 107 | PQBP1 | na | 23052 | -0.066 | 0.0391 | Yes |
| 108 | SNRPB | na | 23129 | -0.067 | 0.0464 | Yes |
| 109 | PPIL1 | na | 23224 | -0.069 | 0.0534 | Yes |
| 110 | PRPF38A | na | 23755 | -0.077 | 0.0490 | Yes |
| 111 | PRPF3 | na | 23919 | -0.080 | 0.0555 | Yes |
| 112 | DHX15 | na | 24004 | -0.081 | 0.0645 | Yes |
| 113 | SNRNP27 | na | 24017 | -0.081 | 0.0756 | Yes |
| 114 | HSPA1L | na | 24075 | -0.082 | 0.0855 | Yes |
| 115 | SNRPA1 | na | 24248 | -0.085 | 0.0925 | Yes |
| 116 | BCAS2 | na | 24404 | -0.088 | 0.1004 | Yes |
| 117 | TCERG1 | na | 24424 | -0.088 | 0.1123 | Yes |
| 118 | SRSF7 | na | 24543 | -0.090 | 0.1217 | Yes |
| 119 | SNRPD3 | na | 24607 | -0.092 | 0.1328 | Yes |
| 120 | SMNDC1 | na | 24715 | -0.093 | 0.1429 | Yes |
| 121 | SNRPC | na | 24868 | -0.096 | 0.1520 | Yes |
| 122 | RBMX | na | 25720 | -0.111 | 0.1432 | No |
| 123 | SYF2 | na | 29529 | -0.211 | 0.0629 | No |
| 124 | HSPA6 | na | 31406 | -0.289 | 0.0495 | No |
| 125 | SF3B4 | na | 32117 | -0.329 | 0.0755 | No |
Table: GSEA details [plain text format]

  

Fig 2: KEGG\_SPLICEOSOME      
 Blue-Pink O' Gram in the Space of the Analyzed GeneSet

  

Fig 3: KEGG\_SPLICEOSOME: Random ES distribution      
 Gene set null distribution of ES for **KEGG\_SPLICEOSOME**
